# A qualitative study of older adults seeking appropriate treatment to self-manage their chronic pain in rural North-East Thailand

**DOI:** 10.1186/s12877-015-0164-3

**Published:** 2015-12-14

**Authors:** Ladawan Panpanit, Mary Carolan-Olah, Terence V. McCann

**Affiliations:** Faculty of Nursing, Khoen Kaen University, Khon Kaen, Thailand; Centre for Chronic Disease, College of Health and Biomedicine (Disciplines of Nursing and Midwifery), Victoria University, PO Box 14428, Melbourne, Victoria 8001 Australia

**Keywords:** Pain, Chronic pain, Self-management, Older adults, Poverty, Healthcare, Thai

## Abstract

**Background:**

Many older adults suffer from chronic pain which decreases their functional capacity and reduces quality of life. Health behaviours and self-care during chronic illness and chronic pain can exert an important influence on health outcomes. The aims of this study were to (a) understand how older adult Thai individuals seek appropriate treatment to self-manage their chronic pain, and (b) to identify factors that contribute to effective pain self-management.

**Methods:**

Qualitative interviews were conducted with 32 older adults living in villages in north-east Thailand. Observations were also conducted with consenting individuals. Most interviews were audio-recorded and transcripts were coded and analysed using a grounded theory approach.

**Results:**

Six contextual determinants affected the way participants choose to self-manage their chronic pain, including: priority accorded to pain management; information and resource seeking skills; critical appraisal skills; access to pain-related information; access to treatment; and satisfaction and preferences for practitioners. Participants used several strategies to inform and develop their self-management plans: accessing and responding to information, sourcing resources, trial and error, evaluating treatment and evaluating practitioners.

**Conclusions:**

Attempts to increase accessibility, affordability and acceptability of pain treatment can promote pain self-management in older Thais. These findings have important implications for health professionals and government organisations seeking to enhance the self-management of pain and quality of life in this population.

## Background

Older adults constitute a growing proportion of the Thai population [1], and chronic pain is an increasingly common health problem among this group [2, 3]. The Thai health policy recognises this issue [4] and there is consensus on the importance of improving older adults’ knowledge about self-management of chronic conditions, and providing them with the means to facilitate self-care [5].

Self-management plays a vital role in maintaining well-being among individuals with chronic illness [6, 7] and key factors associated with effective self-management include: accessible health care services [8] and adequate physical, psychological, material, transportation and financial resources [9, 10]. In the Thai context, studies indicate that older adults’ health behaviours also exert an influence their well-being [11, 12], particularly self-care before and during illness [13]. However, it is clear that many Thais lack appropriate knowledge and skills to self-manage their conditions [14]. This dilemma, of limited knowledge and skills, is compounded by distance from services, particularly for remote and rural communities (Liamputtong [15]).

Older adults living in rural communities in north-east Thailand are the subject of interest in this study, and most have limited access to health care services and other resources [1]. These features reduce their capacity to self-manage their chronic pain, as self-management requires knowledge of health problems, adequate services, and interactions that promote self-management [16, 17]. Chronic pain is identified as an increasing health problem among older adults living in rural communities in north-east Thailand. Nonetheless, little is known about self-management of chronic pain in this community. Understanding how these older adults deal with chronic pain is important in terms of tailoring services to effectively promote self-management strategies and maintain well-being [10]. In this study, we sought to understand how older Thai adults living in North-east Thai communities self-manage their chronic pain within their social-cultural and psychological contexts.

## Methods

### Design

Corbin and Strauss’ [18] approach to grounded theory methodology was used to guide data collection and analysis. The epistemological assumptions of grounded theory are based on symbolic interactionism, in which interactions between individuals’ social roles and behaviours are examined [19]. Emphasis is also placed on identifying social problems and processes [20]. Within the context of the current study, a substantive theory was abstracted relating to the strategies older adult Thais used to self-manage their chronic pain. However, in this paper, we focus only on the strategies participants adopted, and the contextual factors that contributed to their effective pain self-management.

### Ethics

Victoria University Human Research Ethics Committee, Melbourne and the Ethical Review Committee for Research on Human Subjects, Ministry of Public Health, Bangkok, Thailand gave permission to conduct the study. All participants provided voluntary, written consent.

### Selection and recruitment of participants

Participants were recruited from three villages (Village A, B, C), from separate provinces (Roi-Et, Maha Sarakham and Khon Kaen) in the north-east region of Thailand, which is the poorest region in the country [21]. Older adults made up 11 % of the population in these remote regions in 2009, which is an increase of approximately 2 % on 2000 figures [22]. For participating villages, the nearest health centres were located at some distance, in the villages’ sub-districts. All 3 villages were relatively isolated from their provincial capitals (40kms, 45kms and 80kms respectively). Dilemmas associated with isolation were compounded by poor availability and access to public transport.

Recruitment proceeded in the following manner. Initially, brief information about the study was provided to potential participants by public health care centre staff, village health volunteers, or formal/informal leaders of each village. Contact details of prospective participants who expressed interest in taking part in the study were forwarded to the researcher, who followed-up to provide more information and answer questions about the study, and obtain consent. Purposive sampling, or sampling using certain predetermined criteria [23], was used at the outset to inform data collection. The *inclusion criteria* were: male or female Thais; aged 60 years or over; experiencing chronic pain for at least 6 months; living in the selected villages; and ability to communicate in conversational Thai or north-east Thai dialect. The *exclusion criteria* included: mental confusion due to illnesses such as dementia; currently experiencing an acute and serious medical illness or acute pain. All documents relating to the study (e.g., Participant Information Form, Consent Form) were translated into Thai, in accordance with the WHO translation guidelines [24]. The documents were translated into Thai by a bilingual native Thai and then back translated into English by a bilingual native English-speaking person.

Further data collection was based on theoretical sampling, where decisions about sampling participants, setting and type of data collected were founded on the emerging theory [18]. Theoretical saturation of the main concepts abstracted from the data governed the final number of participants and duration of time spent in the field [23].

### Procedure

Simultaneous data collection and analysis, an essential part of the grounded theory approach, occurred [18]. Two main methods of data collection were used: interviews and observations. Interviews were semi-structured, audio-recorded and guided by an aide-memoire or interview guide. Handwritten field notes were also used. For some participants, more than one interview was carried out in order to further explore issues emerging in the data. Interviews took place in a range of settings; for example, in or around participants’ homes and elsewhere in the villages. Overall, 58 interviews (32 in-depth and 26 brief interviews) were undertaken, ranging in duration from 20 to 70 min, in Thai or north-east Thai dialect.

Participant observations were used to collect data and generate theoretical accuracy, which was grounded in the social reality of participants’ everyday lives [25]. Eight observation periods, ranging in duration from 15–120 min, took place in various settings, in participants’ homes, a health centre, a private clinic, and in a private and a public hospital. The researcher adopted an ‘observer as participant’ role [26], with Spradley’s [27] framework being used to guide the observations. Observation periods included observing participants receiving a massage from a traditional therapist and from a family member; participants administrating a hot herbal compress and preparing boiled herbal medication; and four discrete observations of participants consulting with health care staff/doctors.

### Data analysis

Audio-recorded interviews and handwritten field notes and memos were transcribed, in Thai, after each episode of fieldwork, and were then translated into English. The coding process commenced with open coding, where conceptual labels were applied to the data. Labels were grouped into more abstract categories, and the properties and dimensions of categories were established. In the next analysis phase, axial coding was undertaken, where data were put back together differently, through a process of categorising data and establishing connections between categories and sub-categories [28]. In the final phase, selective coding was undertaken, to identify a core or overarching category. The purpose of this sorting was to ‘weave the fractured story back together again’ ([29], p.72). Initial data coding and category and contextual determinant identification was undertaken by LP, followed by an independent review of the activity [30] by TMcC and MCO, both experienced qualitative researchers. The abstracted categories were discussed until consensus was achieved.

## Results

Thirty-two older adults (6 males and 26 females) with chronic pain participated in the study. Mean age was 72.2 years old, ranging from 60 to 87 years. All were Buddhist and almost half were widowed. Highest level of education was primary education. All belonged to a health insurance scheme, with most covered by the Thai government Universal Coverage Health Scheme. Twenty four participants also suffered chronic medical illness, most commonly hypertension and diabetes mellitus. All participants had localised pain ─ some in several locations ─ and their self-rated pain scores, based on the Numerical Rating Scale [31], averaged 6.3 (ranging from 1–10). The most common sites for localised pain were leg (*n* = 21), lumbar (*n* = 20), knee joint (*n* = 16), and lumbar-to-leg (*n* = 15).

Almost all the participants had a low income, with half receiving the ‘Bia Young Cheep,’ a modest Thai government monthly allowance for needy older adults living in rural areas. For one-third of participants, this was their only source of income. Others indicated that they received no income from work or from the government, and they relied financially on their families. Household incomes of most of the supporting families were also meagre. The main industry in the area was agriculture.

### The context of seeking the most suitable treatment

Six contextual determinants simultaneously enabled and inhibited the way participants sought the most suitable treatment for self-management of chronic pain. Determinants included: the priority given to pain management; information and resource seeking skills; critical appraisal skills; access to pain-related information; access to pain relief treatment; and satisfaction and preferences for practitioners and treatments. Contextual situations were not static however, and when they varied, participants had to modify their approaches to self-managing their illnesses.

#### Priority given to pain

Participants placed differing levels of emphasis on seeking suitable treatment for their pain, based on other competing influences in their lives. Some concentrated mainly on addressing their pain, while others placed greater emphasis on attending to other matters in their lives, such as caring for family members. Those who placed a higher priority on dealing with their pain sought help earlier:

I went to bed at night [I felt well]. When I woke up [in the early morning] …. I could not get up from my mattress …. He [my son] asked me, “What happened to you?” I told him, “I have severe pain in my waist. I cannot walk” … “Mum has waist pain. Accompany me to go to see doctor at the private clinic in … District.” [That morning] I went to see Doctor … in … District. (Interviewee 11)

In comparison, participants who placed more emphasis on dealing with other priorities in their lives delayed help-seeking to self-manage their pain.

I had the symptom [pain]. One of my daughters had a serious illness during my first period of having pain .… After she died … I began to seek treatment for my own illness. (Interviewee 2)

#### Information and resource seeking skills

Participants’ abilities to access information and resources about pain management affected the way they dealt with their pain. Those who had good information and resource-seeking skills, such as the ability to question health professionals, were more likely to obtain additional information and adopt more flexible strategies to manage their chronic pain. One participant explained: “I asked him lots of questions … He always explained to me … I asked him many questions. When I went to Mahasarakham Province, the staff explained things to me very well” (Interviewee 27). Many others, however, were hesitant to seek help from health professionals, and, generally, were uncertain about where to seek assistance for their pain.

I do not tell them [the health care staff at the Public Hospital where I go regularly to have a follow-up appointment for hypertension] about my hip [pain] problem …. I don’t know who can help me with my pain. I will live my life in this pattern [suffering pain] until I die. (Interviewee 30)

#### Critical appraisal skills

Even though all participants had the same level of primary school education, they demonstrated contrasting levels of critical appraisal of treatments and practitioners. Some were eager to try every pain treatment that was claimed to be effective, whereas others were more cautious and asked questions before making decisions:

Mr. S’s wife, who lives in … Village … asked me, “… do you want to take this herbal medication? … Many people in … village have recovered now” … Then I asked myself, “How good is it? If it’s effective, why have many people still got the illness? (Interviewee 1)

Some participants were curious to know more about qualified and lay practitioners, who were reported to be skilful in treating pain.

Some people said that they recovered because of the quack doctors. I went to see the new quacks all the time. The quacks that I had seen already, I did not go to again [because their treatment did not work for me]. (Interviewee 10)

#### Access to pain-related information

Participants’ primary sources of pain-related information were from lay people in their villages, healers and retailers of treatments from other places, radio programmes, and health care providers. Due to short consultation times, participants had less access to information from health personnel than from non-health personnel. This made them uncertain about the cause of, and ways to relieve, their pain: “They [the doctors] did not tell me [what my illness was] …. He did not tell [me what I should do to relieve my symptoms]. He just told me to see him regularly” (Interviewee 5).

Pain-related health information was also provided by health personnel in published, electronic, and broadcast media. However, participants lived in rural areas and had low incomes, and this limited their access to published media. In addition, their low level of education restricted their ability to read such material. None had access to, or were knowledgeable about how to use, the Internet. All had television, but claimed that few health programmes were broadcast through this medium. Radio was another key source of information for participants; however, none reported hearing about pain-related information presented by health personnel on the radio. Only one participant reported hearing an announcement by the provincial health office that the herbal pain remedy she was taking could be harmful. This resulted in her ceasing to take the herb: “People said on the radio that ‘If you take this herb, you will die from cancer. There is a large amount of toxin in this herb. Stop taking it.’ Then, we stopped taking it all over my village” (Interviewee 7).

#### Access to pain relief treatments

Access to pain relief treatments had a major influence on the way participants managed their chronic pain. The most accessible treatments were more likely to be adopted although they felt these were not as effective as some less accessible treatments. Participants commonly used three main treatment types: conventional medicines, traditional medicines or complementary and alternative medicine (e.g., herbs taken orally or applied as a compress, massage, acupuncture, spiritual healing). Most had limited access to conventional treatments provided by health care providers, whereas some over-the-counter conventional medicines were accessible. Overall, participants had greater access to traditional remedies in their villages and this treatment was more affordable. Lay-informed treatments were also commonly adopted.

Several participants commented on limited public resources, which resulted in long waiting times: “At the public hospital … I waited there for 2–3 h … I fell asleep [while waiting] one or two times .… This made me bored [fed up with the public hospital]” (Interviewee 4). In addition to lengthy waiting times, several participants felt these services were a waste of time for themselves and their caregivers. Consequently, most avoided accessing public health services, preferring instead to seek treatment from private, pay-for-use services. There were shortcomings, however, with private clinic services, and participants were critical about lengthy waiting and short consultation times.

I went there, [and I had to wait a long time]. When he [the doctor] came … he ran [was hurrying] to the examination room and his staff told each person to go to see him in the examination room. [When I went into the room], he … just used the stethoscope to touch my body, and then he gave me an injection and told me that I would recover soon … [He said,] “just take these oral medications.” (Interviewee 23)

Other factors contributed to participants’ difficulties in obtaining access to treatment, including a social belief that pain is a normal phenomenon in older adults, and a higher risk of serious complications associated with treatment of older adults.

People say that it [my pain] is because I am elderly. It might not be cured. At the hospital a doctor said …. “They [your joint pain and being older] are together for a long time. When we are old [we get pain].” (Interviewee 12)

#### Satisfaction with, and preferences for, practitioners and treatments

Satisfaction with treatment received earlier, influenced subsequent help-seeking. As described previously, many participants were dissatisfied with conventional treatments received from public and private health care services and sought alternative services such as traditional remedies and over-the-counter medications. Dissatisfaction with pain relief treatments was expressed in three main areas: perceived impractical suggestions about pain management, inadequate pain relief, and unclear information being provided at consultation. Participants sometimes found that the treatment they considered most effective was discouraged by health personnel, who then offered no effective alternatives. Some reported experiencing more obvious adverse effects from the treatment provided by health personnel than from treatments they were instructed to avoid.

When I went [to see the doctor to discuss] for the operation, he told me, “Stop taking the black and red herbal tablet, grandma. There are many people [who have taken the tablet] who have died already.” … When I stopped taking the herbal tablet, I felt pain a lot. There was much swelling [in my joints] … He explained to me and wanted me to stop taking it. Then he gave me some oral medications. I took his medications, and I vomited a lot … I felt that I was near to dying. (Interviewee 27)

Many participants indicated that the services they received from health personnel did not meet their needs for self-managing chronic pain, prompting them to find other ways to deal with their pain.

When I asked for the ointment [from the health centre staff] I received only one tube. I think one tube can be applied for one time only because I apply it from my feet up to my waist now [And I have to buy the ointment from other places to apply for my pain]. (Interviewee 27)

Others felt that the treatments provided by health personnel were inconsistent with the cause of their chronic pain. They expected these personnel to provide more specific treatments for the underlying cause of their pain.

If I have kidney disease, I want them [the doctors] to give me the medications for kidneys. If they said that I have calculi, I want to take the medications for calculi … I want them to give me the right medications, not only the medications for peptic ulcer. I know the medications. I have taken the medications many times. Don’t just only give me the same medication for all different illnesses I suffer. (Interviewee 25)

### Strategies for seeking the most suitable treatment

Within this backdrop of the contextual determinants, participants used five main strategies for *seeking the most suitable treatment* to self-manage their chronic pain: gathering and responding to information, accessing resources, using trial and error, evaluating treatments, and evaluating practitioners. These strategies focused on how participants obtained, assessed, changed or maintained their pain management behaviour. However, once a contextual situation changed in their lives, this necessitated a modification in their approach(es) to pain management.

#### Gathering and responding to information

Generally, participants had limited access to written information and sought information was by word-of-mouth and communication with lay people. One participant explains: “… told me that she had pain. She went to see doctors in many places, but they could not relieve all her pain. She applied this balm and she felt no pain” (Interviewee 7). In addition, some participants learnt about pain treatments by observing the effects of these treatments on other people.

My eldest sister … she said she felt pain if she stopped taking it [the herbal tablet]. When she took it, she could go to the temple, and she did not stop taking it. [And she has already died from its side effects]. (Interviewee 7)

The most common method of obtaining information from health personnel was by engaging in passive one-way communication, listening to their suggestions. “They [the doctors] told me, ‘Grandma, take this medication. You cannot recover whatever you do. You can only relieve your symptoms to some degree” (Interviewee 10). Some participants sought to extend their understanding by asking questions but the responses they received did not always satisfy.

I asked, “Why will doctors only cut [amputate] my legs?” [The doctor did not answer my question but said to me], “So, take a rest and, grandma. Take a rest like this. Wait until the legs are not swollen. Then we will do what we will do.” (Interviewee 2)

Many considered injections to be powerful at relieving pain and were disappointed when this form of pain relief was unavailable

I told him [the health centre staff], “Could you give me an injection? I have extremely painful [legs].” He replied, “Does your pain come because you walk too much? .… (Interviewee 5)

They also considered the feasibility of adopting pain relieving suggestions to their individual circumstances, including financial.

The doctor at … [private] hospital said such words as, “It [your pain] cannot be cured if you do not have the operation.” I do not have money to go [for an operation] …. They suggested me to have an operation, but I thought I am already old; I would just let it go [not have the operation]. (Interviewee 30)

Several participants were cautious about the information they received and consulted with other people, especially family members, before deciding whether to take a particular treatment.

He said, “It’s a herb.” He fermented it. It has to be fermented for three months …. Then I called my grandchildren to ask their opinion. They said, “Even the experts [doctors] say that the chance [of recovering from your pain] is 50/50, grandma. Please do not take it. Medicine that we have never seen [before], we never know. (Interviewee 1)

#### Accessing resources

In addition to information resources, participants accessed additional resources to help them manage their pain, including treatments, financial and transportation support. The ways in which they obtained these additional resources ranged from seeking assistance from others, which involved little effort, to intense seeking of pain relieving medications, which required considerable effort: “People all over the village [who suffered pain] drank it [the herb’s liquid after boiling]. We went to find it no matter how far away it was …. I dry it and keep it in this big bag” (Interviewee 7). Several participants sought assistance from family members, to obtain or enable them to use treatments.

I always ask my children [to accompany me] to go [to seek pain treatment] … “Let’s accompany me to go [to see doctors]. [let’s take mum to] go to this place, go to that place,” I said to my children …. Wherever people say it’s good, I ask my children to take me [there]. (Interviewee 2)

Financial support was another resource participants sought. Most asked for money from their children to enable them to pay for treatment: “If [I] have no money, I ask for [some] from him [my son]” (Interviewee 13). They also sought assistance with transport, particularly when providers were located far away: “My husband accompanies me to go [to the health centre] in a wheelchair” (Interviewee 20).

#### Using trial and error

Once participants considered particular treatments might be effective for their pain, they used trial and error to evaluate the treatments until the most appropriate method of pain management was identified. More than half the participants used conventional treatments as their preferred method of pain self-management. However, most who tried initially only conventional treatments reverted to using traditional treatments and/or their own lay-informed approaches. Two participants who began their pain treatments with conventional medicine continued to use this style of treatment only, but modified their treatments and health practitioners within that style.

I went to see Doctor … [at his private clinic] …. Then I went to … [public] hospital … I went to Khon Kaen [public hospital] …. Now I go to see the doctor only at the private clinic in … District [a district in an adjacent province]. (Interviewee 11)

Many also used traditional medicine when they first experienced pain, using it in combination with conventional treatment and/or lay-informed approaches.

At the beginning of my pain, I went wherever I heard that there was good management for pain ….. I went to see only traditional therapists … I went to … District, Khon Kaen, and … Village …. Only the person in … District told me to avoid eating the forbidden foods. He told me, “Grandma, this disease cannot be cured no matter who has it” … I took the medications prescribed by Doctor … (Interviewee 14)

Lay-informed approaches were used least when participants began suffering pain, but later become the most common approaches during the final trial and error steps.

I boil many kinds of herbs that I can take from around my house and drink their liquid. I also exercise regularly. These two things help relieve my pain, and I haven’t used any other type of pain treatment. (Interviewee 15)

These lay-informed approaches were much less costly than other approaches, easy to access or adopt, convenient to use, and brought some relief to their pain.

#### Evaluating treatments

Participants used several interconnected criteria for assessing treatments. They assessed them for their cost-effectiveness, discomfort/presence of side effects, and for convenience of access and use. Most reported that while the treatments helped relieve their pain temporarily, the pain reoccurred once the analgesic effect diminished.

I decided to stop taking the medications [because] I did not recover after taking them. After the [analgesic] effect of the medication had gone, my pain returned…. The doctor’s treatment did not work for me. The same thing occurred when I had injections from the doctors. (Interviewee 19)

Some found that medication did not change the severity of their pain. Several commented that their favourite remedies made them feel better than other treatments, whereas most reported that treatments made their pain more tolerable: “I take this kind of medication [Ibuprofen] and it helps my pain to be tolerable …. I feel better. The feeling of stiffness in my joints also decreases” (Interviewee 24). Some stated that treatments from public and private health care practitioners were equally effective. Alternatively, some commented that treatments obtained from private health care settings were more effective than those from public facilities.

People said, and I think it is true: when people have a mild illness and go to see the doctor there [at the private hospital] .... they recover. [Doctors in] the private hospital give effective medication to get rid of the disease. (Interviewee 5)

The cost incurred in obtaining treatments was another evaluative criterion. Some treatments, although effective, were discontinued because of high costs.

The cost per month will be many thousands or tens of thousands [of baht], if people take it everyday … It is expensive … If the price is 50–60 baht (US$1.5–1.8) per bottle, it would be okay for me. But, it is 500 baht (US$15.3) a bottle. (Interviewee 5)

Participants also assessed the cost-effectiveness of treatment, commenting that while some remedies were expensive, they were willing to pay for them if the treatment was effective in managing their pain.

I bought it for 500 baht (US$15.3) …. If I felt better … I would find [borrow] some money to buy the medication even though I don’t have money myself. But I feel the same as before. I say so due to the fact that I don’t feel better [after taking the medication]. (Interviewee 5)

Treatment-related side effects were also evaluated. Intolerable and severe side effects led some participants to discontinue a treatment, while others persevered with treatments if they were informed in advance that adverse effects would diminish eventually.

Convenience in using and accessing treatments was another criterion. Some effective treatments were discontinued as a result of inconvenience: “I boiled and took its [a flower villagers obtain from the paddy fields] liquid to relieve pain two times … I stopped taking the flower. I am not diligent. Yes, I am lazy to boil the flower” (Interviewee 29). Participants compared treatments for accessibility and general user friendliness. Most emphasised that it was more convenient to go to private than public health care settings because of the problem of slow service and long waiting times at public services.

The private hospitals were different from public hospitals. What is the difference? …. Why do people go [to private hospital] even though they have to pay much money? It is convenience .... For the public hospital, [when people] go to this room, [they] have to wait for two hours, in … Hospital … I went there once with … [my son]. I waited there for two or three hours …. (Interviewee 4)

#### Evaluating practitioners

Participants also evaluated practitioners who provided treatments. Evaluative criteria included knowledge and skills related to treatment, behaviours while delivering treatments, concerns about participants’ well-being, and moral principles. They assessed conventional and traditional practitioners’ knowledge, qualifications, and treatment procedures.

I looked around there [the practitioner’s clinic] … I just wanted to know from where he graduated. There was nothing [no certificate showing his qualification] there. There were only bottles of medication, a bed, and a wide floor. He gave injections [while people were lying] on the bed. He used only hot water [to clean the needle]. And I thought, “[He was] a sub-standard doctor!” (Interviewee 26)

In addition to assessing practitioners’ knowledge, skills, and behaviours while giving treatments, participants were sensitive to the concern practitioners showed for their well-being. The following account is illustrative:

The nurse there [at the hospital] also treats me well. When I go to receive the medications, she holds my hand to go and said to me, “I afraid that you will forget to come.” She is also nice to me …. (Interviewee 27)

The final criterion participants used to assess treatment were cultural and moral principles. Based on their cultural beliefs, they presumed that some traditional practitioners had moral integrity and provided treatment with the intent of helping others. This included consideration of the participants’ financial circumstances:

There are so many people who go to see him [the traditional therapist] and he has a bowl for these people to put the money [in to pay] for his treatment. The amount of money given is up to clients. I asked him, “Did I have to pay only 20 baht (US$0.6)? Why is it so little?” He replied, “With only this amount of money [per person], I will be rich very soon [because lots of people come to receive my treatment]” …. Why do I have to charge you much money? Your travelling costs are already expensive,” he said. (Interviewee 2)

## Discussion

In this exploratory study, we sought to understand the contextual influences and the strategies older Thais adopted to self-manage their pain. The findings indicate three main considerations for participants: accessibility, affordability, and acceptability of treatment. These considerations were affected by the context of participants lives, which, in turn, influenced strategies they adopted in seeking the most accessible, affordable and acceptable treatments for their chronic pain. Our findings are consistent with the findings of a study by Goudge et al. [32] who found that factors moderating help-seeking in all age groups included: affordability, availability and acceptability. Similarly, a Thai study by Petkong [33] that suggested five factors influenced help-seeking from health services; namely, availability, accessibility, accountability, alternatives, and acceptability.

The choice of pain treatment was influenced primarily by participants’ access to treatments, which was limited because of poverty and rural isolation. The term ‘multi-faceted vulnerability,’ coined by Radley, Hodgetts and Cullen [34] which refers to vulnerability due to factors which diminish autonomy and marginalise life [15], may offer some explanation for participants’ experiences in this study. Similar to Liamputtong’s (2007) description, five factors contributed to the vulnerability of participants in the current study: their advanced age, chronic illness, rural residential location, region of the country, and poverty [15]. Within these limitations, participants often made choices based on access and affordability rather than perceived effectiveness of the treatment. Traditional medicine, a common form of treatment in Thailand [35], played a vital role in their participants’ choices. This form of treatment is generally affordable and available locally. Moreover, its adoption is founded on Thai cultural beliefs, based on spiritual considerations, and Brahma and Buddhism [36], making it more culturally acceptable. In sum, participants in this study indicated a preference for treatments that were cost-effective, accessible and convenient to use, and this is similar to findings of an earlier literature review of older adults’ approaches to chronic pain self-management [25].

The cost of treatment had adverse implications for access in the present study, and this is an issue that requires further consideration. The Thai Universal Coverage Health Scheme has helped decrease health expenses; nonetheless, it was insufficient to meet the needs of participants. Many resorted to visiting private clinics but later ceased taking prescribed treatments because of high costs. Thus, the cost of medication was a factor in some participants’ decision to choose traditional instead of conventional medicines. This finding contrasts with that of a study of older adults in Taiwan, where conventional medicine was used more frequently than traditional approaches. This difference may be explained in part by the different economic circumstances in the two countries. In Taiwan, conventional medicines are less costly than traditional medicines because the National Health Insurance scheme in that country covers most of the cost [37].

Other factors negatively influencing access to treatment in the present study were excessive waiting times for appointments, concerns about the quality of the public health service, and the cost of transportation. Participants often opted to use private clinics because of long waiting times and uncertain service quality under the Universal Coverage Health Scheme. Additionally, waiting times for medical consultations between public hospitals and private hospitals and clinics in Thailand differ markedly, at approximately 83, 23, and 18 min, respectively, as described in a recent study by Pongsupap & Lerberghe, [38].

Although the Universal Health Coverage Scheme has helped reduce the burden of chronic pain expenditure for participants, there were often other considerable costs involved, particularly transportation, which was an inhibiting factor in accessing care, in this study.

Satisfaction with clinicians and the quality of treatment provided also exerted an influence on participants’ choices. Treatment was chosen if it was considered effective, user-friendly, and had acceptable, tolerable, and/or manageable side effects. Some participants found traditional medicine to be more effective than conventional medicine. In addition, some treatments were regarded as unsuitable because they were too complicated to use, highlighting the importance of providing pain treatment with user-friendly properties. Even though herbal tablets and over-the counter medications containing steroids are widely regarded as dangerous medicines in Thailand [39], they were identified as the preferred method of pain treatment by several participants in the current study. This preference may have been attributable, in part, to the medicine’s effectiveness in reducing their pain and, hence, their toleration of its potentially harmful side effects. Pain treatments producing troublesome side effects were more likely to be tolerated if participants knew in advance that side effects would diminish eventually. This highlights the importance of providing adequate information about treatment side effects when providing prescriptions. Some severe side effects were considered tolerable to participants, irrespective of whether they were informed about them in advance, because the treatment helped relieve their pain.

When assessing practitioners’ attributes, participants in the present study preferred those who they considered trustworthy, who provided continuity of care and were respectful. This finding is consistent with those of a study of African American women in the United States [40], where provider attributes desired by patients included good quality communication, continuity of care, being treated with respect, and compassionate care. Such attributes are generally understood to enhance patient satisfaction [41]. Participants in the present study expressed satisfaction with practitioners who showed concern about them and who listened to their problems about pain self-management. However, these attributes were more evident in traditional than in conventional health practitioners. This finding highlights the importance of patient satisfaction with chronic pain treatment [42] and improved participation in daily activities [43].

A final consideration is the role of families in enhancing self-management in seeking and using treatments. The data indicate that families made varying financial contributions toward the cost of treatment; consulted with participants about treatments, when requested; brought them to consultations; and helped apply some treatments. The influence of families on self-management is not so clear however, in the literature. Some researchers have suggested that family and social network support enhances self-management [44–46], while others have indicated that this form of support can facilitate or hinder self-management [47–50].

### Limitations

Our study has two limitations. As this was a qualitative study, the findings are pertinent to the participants and the context in which it was undertaken. Even though generalisability is not achieved from sample representativeness, the concepts are still applicable to older adults with chronic pain in similar circumstances [51, 52]. In addition, more women than men participated; therefore, the findings might be less applicable to men. The reasons for the greater participation of women included a greater willingness to participate and there was a higher proportion of older women than men in the villages.

## Conclusions

Our study’s findings highlight important understandings about the ways older adult Thais seek to self-manage their chronic pain. The findings have implications for health professionals, government organisations and health policy makers seeking to enhance self-management of pain by older adults. In particular, greater attention needs to be paid to increasing accessibility, affordability and acceptability of pain treatment. For example, consideration should be given to providing older adult Thais living in rural settings with better access to evidence-based information about pain treatments through television and radio media and other initiatives such as volunteer health workers and health centres. Implementing strategies and policies to support self-management of chronic pain in this population may help ensure better control of their chronic pain, enhance their overall well-being, and reduce the economic burden of chronic pain management on health care services. The study findings also have implications for older adults living in these and similar settings and dealing with chronic pain. Providing guidance on effective self-management of pain may support well-being in similar older communities.
